# Racemic *cis*-bis­[bis­(pyrimidin-2-yl)amine-κ*N*]bis(dicyanamido-κ*N* ^1^)iron(II) dihydrate: synthesis, crystal structure and Hirshfeld surface analysis

**DOI:** 10.1107/S2056989023008186

**Published:** 2023-09-26

**Authors:** Yaakoub Saadallah, Zouaoui Setifi, Christian Jelsch, Fatima Setifi, Mohammed Hadi Al-Douh, Achouak Satour, Christopher Glidewell

**Affiliations:** a Laboratoire de Chimie, Ingénierie Moléculaire et Nanostructures (LCIMN), Université Ferhat Abbas Sétif 1, Sétif 19000, Algeria; b Département de Technologie, Faculté de Technologie, Université 20 Août 1955-Skikda, BP 26, Route d’El-Hadaiek, Skikda 21000, Algeria; c Cristallographie, Résonance Magnétique et Modélisations (CRM2), UMR CNRS 7036, Institut Jean Barriol, Université de Lorraine, BP 70239, Boulevard des Aiguillettes, 54506 Vandoeuvre-les-Nancy, France; d Chemistry Department, Faculty of Science, Hadhramout University, Mukalla, Hadhramout, Yemen; e School of Chemistry, University of St Andrews, St Andrews, Fife KY16 9ST, United Kingdom; University of Aberdeen, United Kingdom

**Keywords:** solvothermal synthesis, iron complex, dicyanamido ligands, hydrogen bonding, Hirshfeld surface analysis, crystal structure

## Abstract

The title compound, [Fe(C_2_N_3_)_2_(C_8_H_7_N_5_)_2_]·2H_2_O, has been synthesized solvothermally and characterized by single-crystal X-ray diffraction. The octa­hedral iron coordination polyhedron contains two di(pyrimidin-2-yl)amine ligands coordinated in a bidentate fashion, and two monodentate dicyanimido ligands, each coordinated *via* a terminal N atom, with the latter in a *cis* orientation. The ligand configuration about the iron atom is chiral, although the compound crystallizes as a racemic mixture: the Fe—N distances (> 2.07 Å) are characteristic of high-spin iron(II). In the crystal, an extensive series of N—H⋯N, O—H⋯N and O—H⋯O hydrogen bonds links the independent mol­ecular components into a three-dimensional framework. The H atoms of both water mol­ecules are disordered. The structure also features some π–π and anion–π inter­actions. The inter­molecular inter­actions were investigated by Hirshfeld surface analysis and two-dimensional fingerprint plots. Comparisons are made with some related compounds.

## Abstract 2

The solvothermal synthesis, crystal structure and Hirshfeld surface analysis of a new iron(II) complex containing dicyanamido and di(pyrimidin-2-yl)amine ligands are reported

## 1. Chemical context

Spin crossover (SCO) can occur for some transition-metal complexes where the metal ion is in one of the configurations *d* ^4^, *d* ^5^, *d* ^6^ or *d* ^7^ in which the spin state can be switched between high-spin (HS) and low-spin (LS) states by an external perturbation such as temperature, pressure, magnetic field or light irradiation (Goodwin, 2004 ▸; Halcrow *et al.*, 2019 ▸). In addition to the magnetic changes resulting from the spin-state switching, this SCO behaviour can be accompanied by structural modifications and changes in the optical properties such as colour changes, making these SCO systems promising candidates for applications such as the development of new generations of memory devices and sensors (Sato, 2016 ▸; Bisoyi & Li, 2016 ▸). For the preparation of these compounds, our strategy is based on the use of cyano-carbanion ligands for designing such SCO materials. These organic anions are versatile and effective for developing mol­ecular architectures with different topologies and dimensionalities, as a result of their ability to coordinate and bridge metal ions in many different ways (see, for example, Gaamoune *et al.*, 2010 ▸; Addala *et al.*, 2019 ▸; Setifi *et al.*, 2017 ▸; Merabet *et al.*, 2022 ▸, Dmitrienko *et al.*, 2020 ▸). 
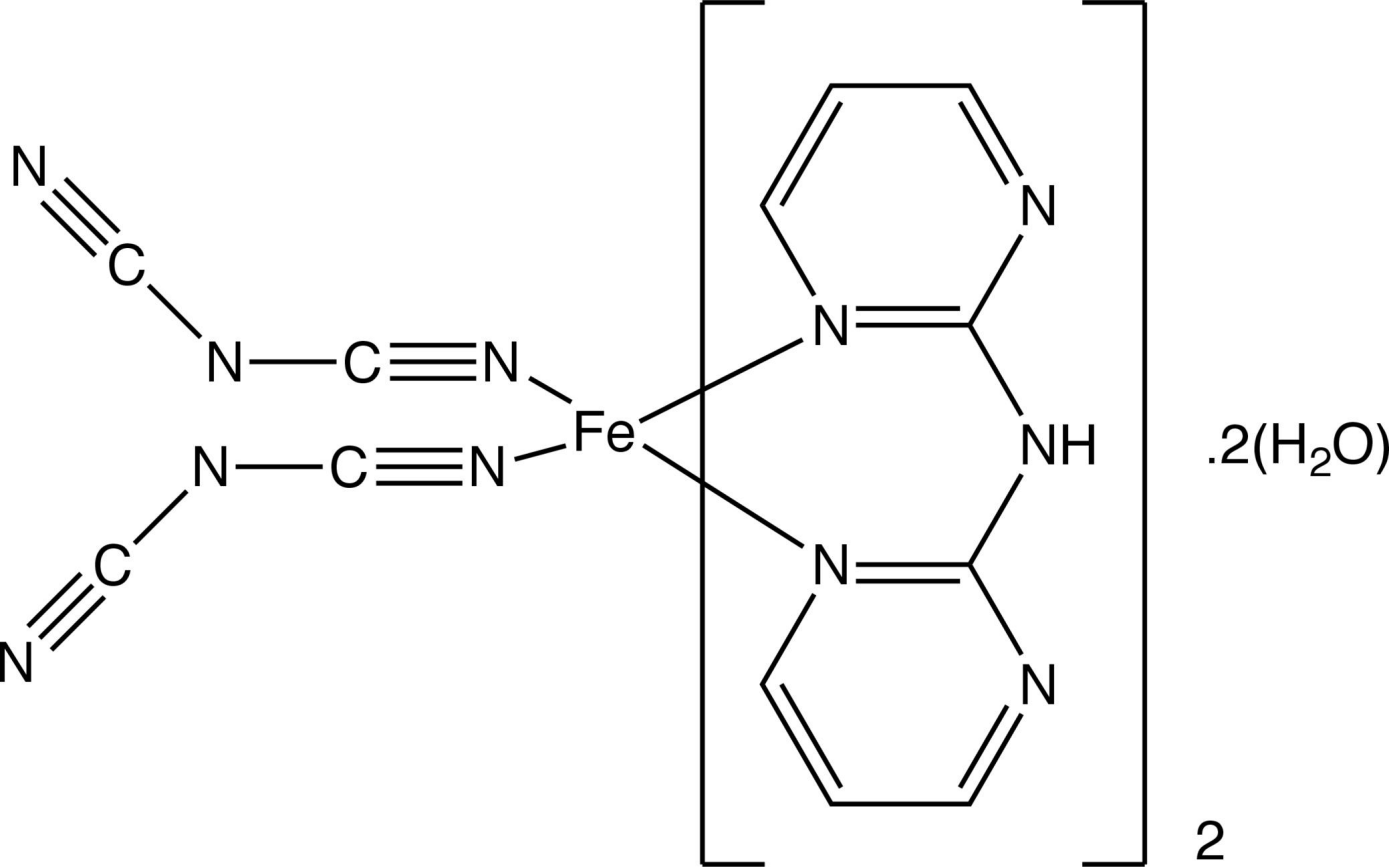


Continuing our study of spin-crossover 3*d*-metal complexes formed by polydentate ligands (Benmansour *et al.*, 2010 ▸; Setifi, Charles *et al.*, 2013 ▸; Setifi, Milin *et al.*, 2014 ▸; Cuza *et al.*, 2021 ▸), we now describe the synthesis and structure of the title Fe^II^ complex, (I), containing the dicyanamido anionic ligand and neutral di-2-pyrimidyl­amine (dipm) as co-ligand, which crystallizes as a dihydrate.

## 2. Structural commentary

**Figure 1:**
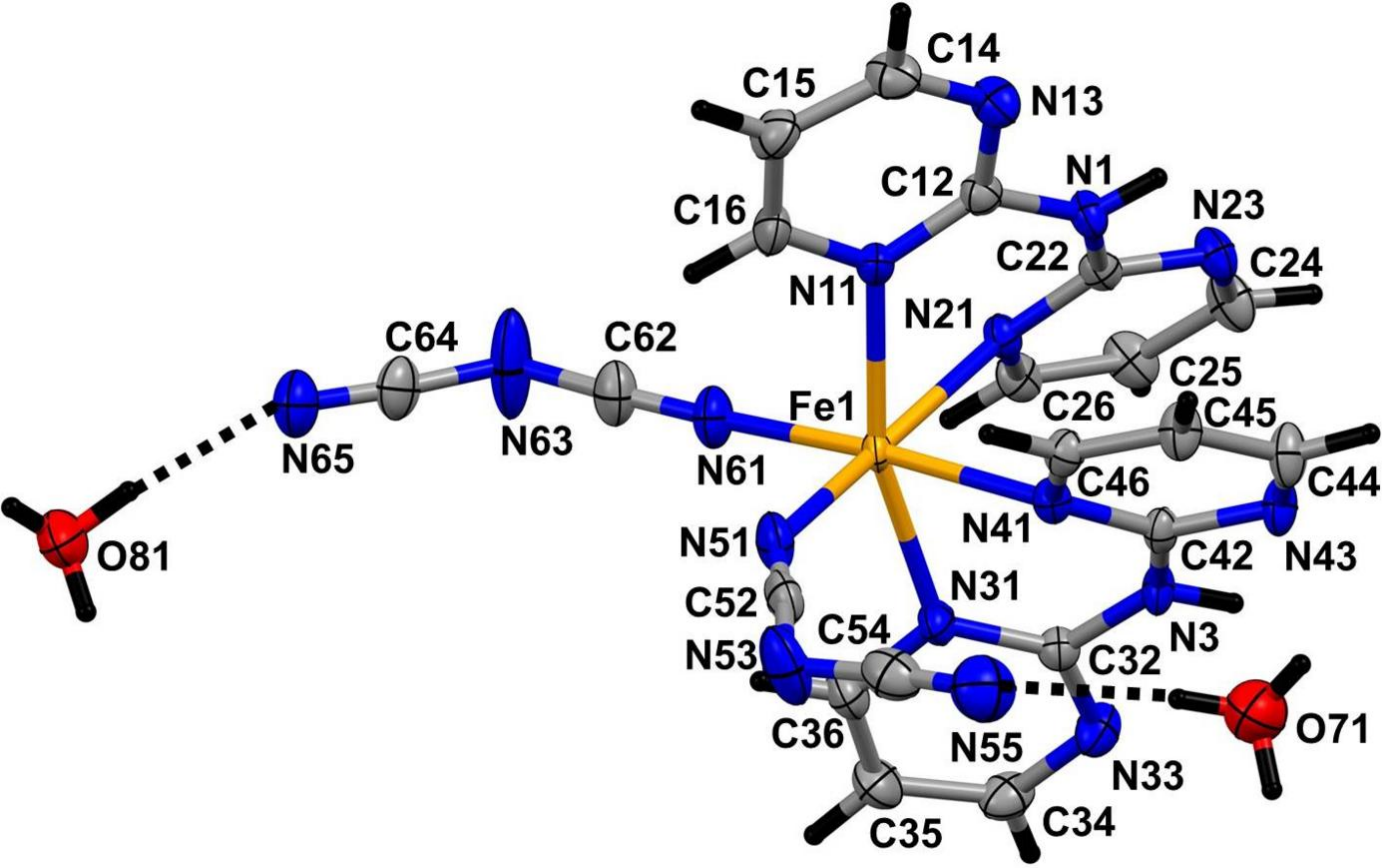
The mol­ecular structure of (I) showing the hydrogen bonds as dashed lines. The water H-atom sites forming the hydrogen bonds to atoms N55 and N65 have full occupancy, but the other water H-atom sites have 0.5 occupancy (see text). Displacement ellipsoids are drawn at the 50% probability level.

In compound (I), which crystallizes as a dihydrate (Fig. 1 ▸), the iron(II) centre is coordinated by two monodentate dicyan­amido ligands, which occupy a pair of *cis* sites, and by two di(pyrimidin-2-yl)amine ligands, each coordinated to the Fe^II^ atom in a bidentate fashion by a pair of pyrimidine N atoms, one in each ring. The complex is thus chiral and in the arbitrarily chosen asymmetric unit, the complex has a Δ configuration, although the centrosymmetric space group confirms that the compound has crystallized as a racemic mixture. Although *cis*-complexes of the general type *M*(*L*–*L*)_2_ *X*_2_, where *L*–*L* represents a bidentate ligand, can exhibit twofold rotation symmetry, that is not the case here, as the two dicyanamide ligands adopt different orientations relative to the rest of the complex (Fig. 1 ▸).

Within the iron complex, the Fe—N distances span the range 2.077 (4)–2.230 (3) Å (Table 1 ▸), indicating that the Fe centre adopts a high-spin configuration at 170 K: for a low-spin configuration, the Fe—N distances would be close to 1.95 Å (Orpen *et al.*, 1989 ▸). In the anionic ligands, there is a marked difference between the central C—N distances, all close to 1.30 Å and the terminal distances, all close to 1.15 Å (Table 1 ▸). Combined with the C—N—C bond angles at the central atoms N53 and N63 of 119.7 (4) and 121.4 (4)°, respectively, these data indicate a strong degree of bond fixation in these ligands, with the negative charge localized primarily on the central N atoms. In this connection, it is inter­esting that the central atoms N53 and N63 participate in neither the hydrogen bonding nor the anion–π contacts (see Section 3, below).

In each of the two independent water mol­ecules, the H atom (H71 or H81) forming an O—H⋯N hydrogen bond (Table 2 ▸) is fully ordered, but the other H atom is disordered over two sites. For the selected asymmetric unit (Fig. 1 ▸), inversion-related pairs of water mol­ecules containing atom O71 lie across the inversion centre at the origin with an O⋯O distances between them of 2.770 (6) Å, and they are linked by half-occupancy H atoms occupying two inversion-related sites separated by only 1.10 Å, so that for any such pair of water mol­ecules, if one site is occupied, the other must be vacant. A similar pair of water mol­ecules containing the atom O81 lies across the inversion centre at (1/2, 1, 1), with the O⋯O separation of 2.751 (5) Å and again linked by disordered H atoms occupying two sites. For the pairs of water mol­ecules containing atom O71, the partial-occupancy H atoms lie close to the O⋯O line, but for the pairs containing atom O81, the OHOH array describes a parallelogram. In addition, the atoms O71 at (*x*, *y*, *z*) and O81 at (−*x*, 1 − *y*, 1 − *z*), are separated by only 2.683 (6) Å and these also are linked by two half-occupancy H-atom sites (Table 2 ▸).

## 3. Supra­molecular features

The structure of compound (I) contains N—H⋯H, O—H⋯N and O—H⋯·O hydrogen bonds (Table 2 ▸) and together these link the independent mol­ecular components into a three-dimensional network. The structure contains no C—H⋯π hydrogen bonds but π–π stacking inter­actions and short anion–π contacts are both present.

**Figure 2:**
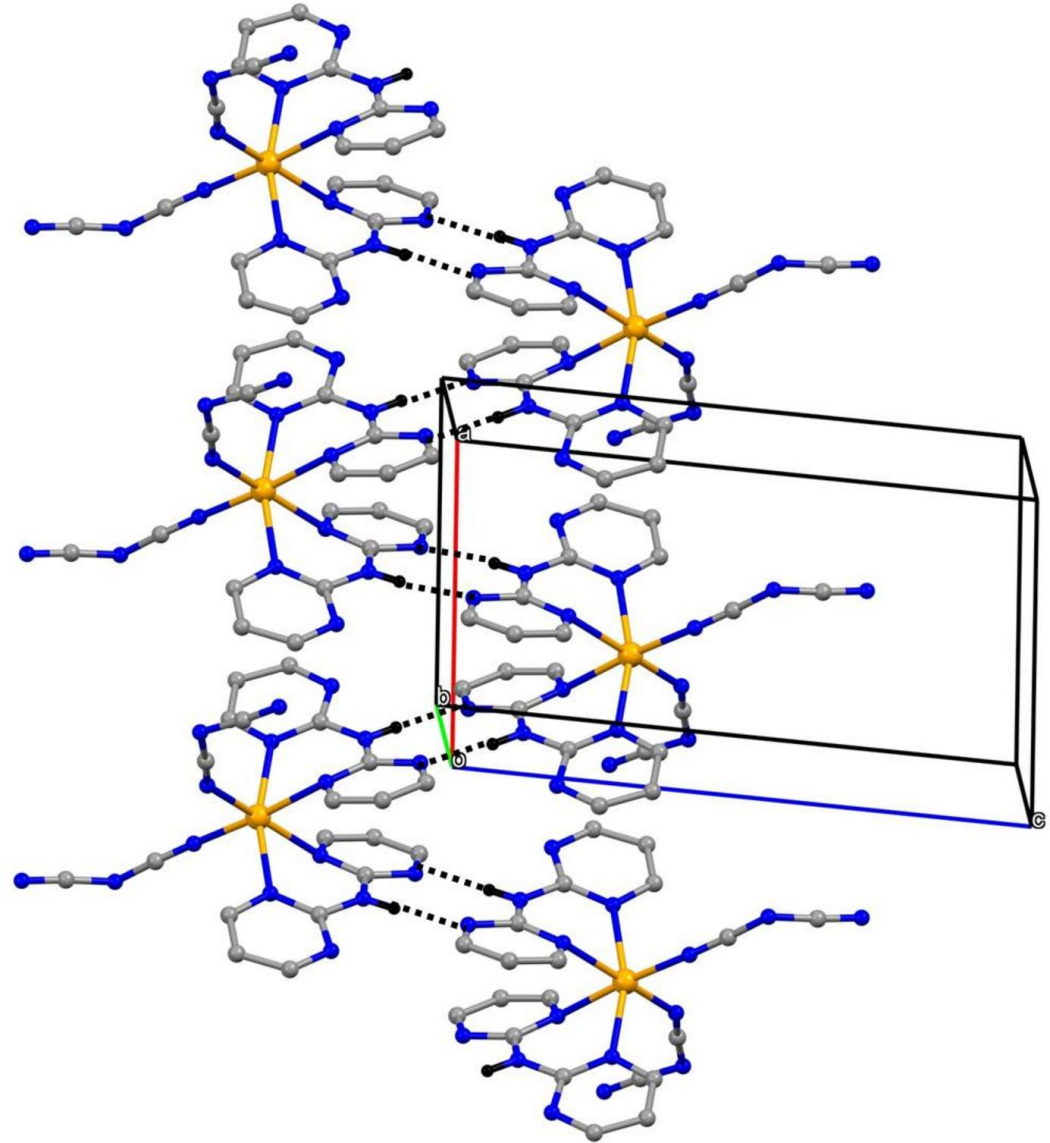
Part of the crystal structure of compound (I) showing the formation of a hydrogen-bonded chain of 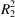 (8) rings formed by the Fe complexes and running parallel to the [100] direction. Hydrogen bonds are drawn as dashed lines and, for the sake of clarity, the water mol­ecules and the H atoms bonded to C atoms have been omitted.

The hydrogen-bonded framework structure is readily analysed in terms of simple one-dimensional substructures (Ferguson *et al.*, 1998*a* ▸,*b* ▸; Gregson *et al.*, 2000 ▸). In the simplest of the sub-structures, the iron complexes are linked by N—H⋯N hydrogen bonds (Table 2 ▸), forming a chain of rings running parallel to the [100] direction (Fig. 2 ▸). Centrosymmetric 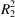 (8) rings (Etter, 1990 ▸; Etter *et al.*, 1990 ▸; Bernstein *et al.*, 1995 ▸) in which atoms of type N1 act as the hydrogen-bond donors are centred at (*n* + ½, ½, 0) and these alternate with 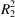 (8) rings in which atoms of type N3 act as the donors and which are centred at (*n*, ½, 0), where *n* represents an integer in each case.

**Figure 3:**
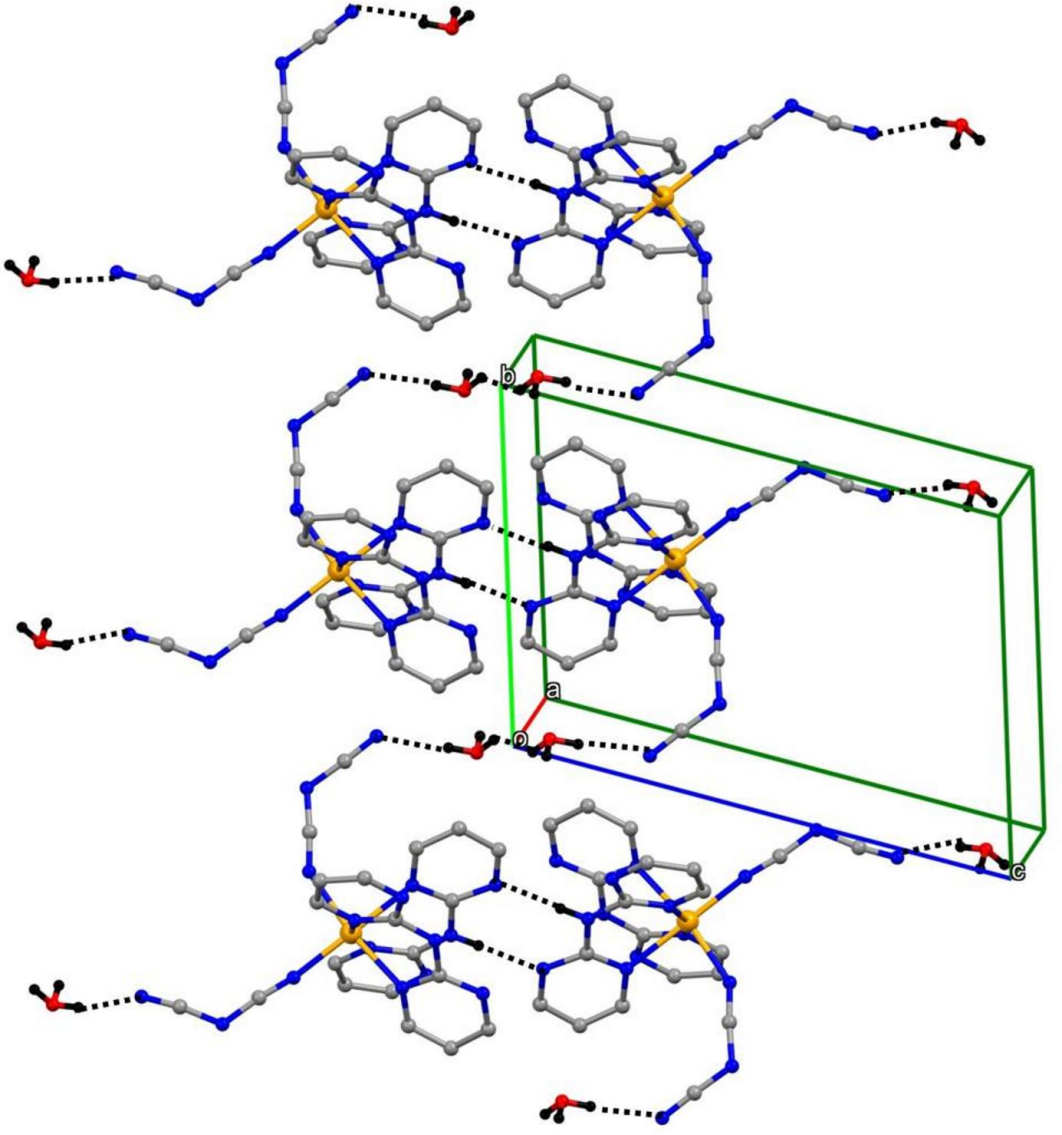
Part of the crystal structure of compound (I) showing the linking of the iron complexes by the water mol­ecules to form a chain parallel to the [010] direction. Hydrogen bonds are drawn as dashed lines and, for the sake of clarity, the H atoms bonded to C atoms have been omitted.

**Figure 4:**
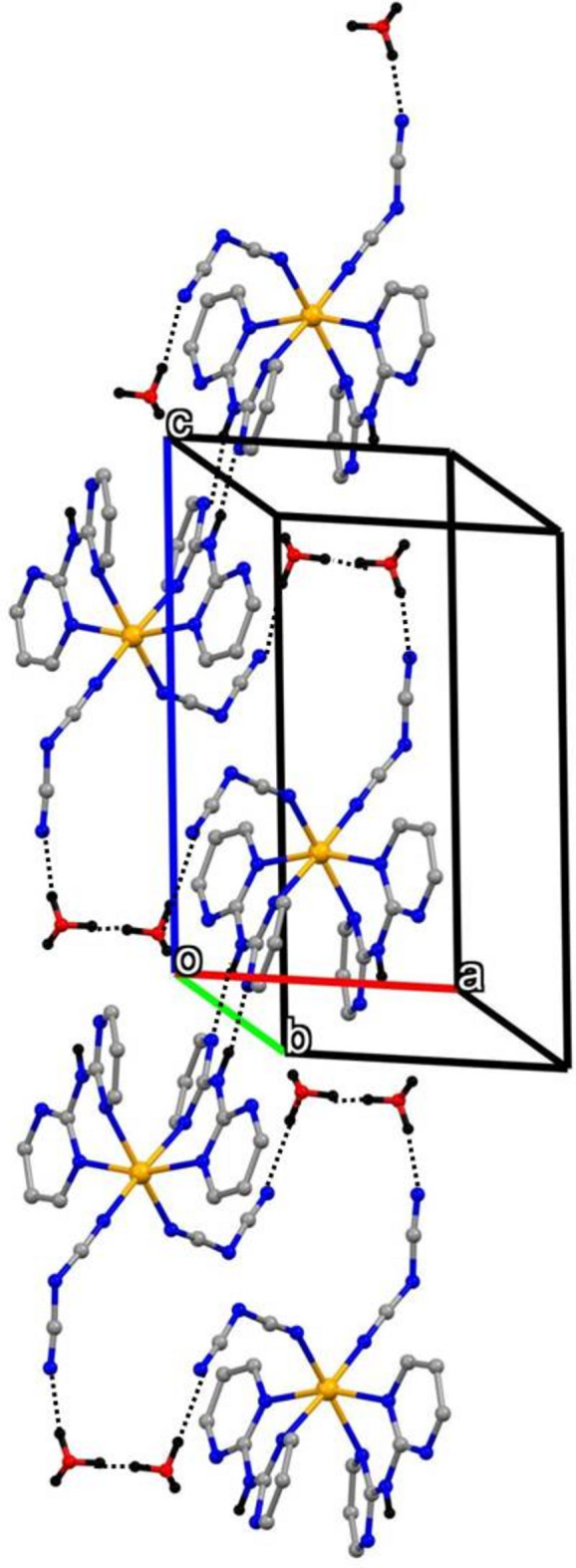
Part of the crystal structure of compound (I) showing the linking of the iron complexes by the water mol­ecules to form a chain parallel to the [001] direction. Hydrogen bonds are drawn as dashed lines and, for the sake of clarity, the H atoms bonded to C atoms have been omitted.

**Figure 5:**
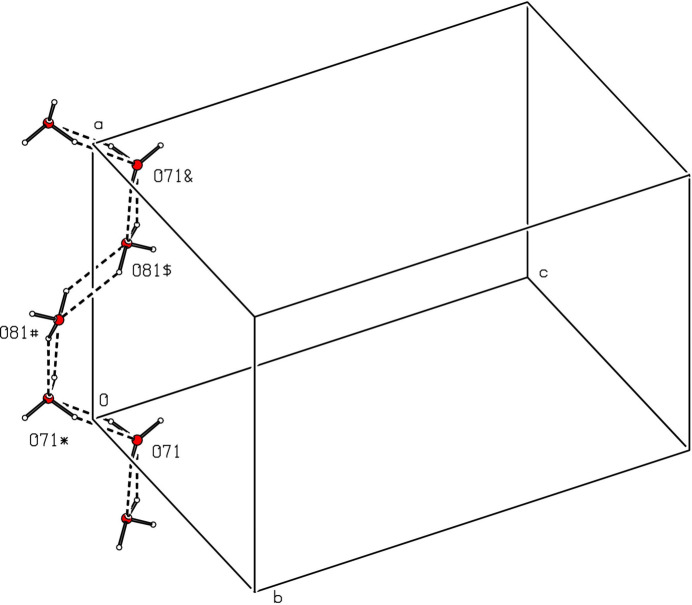
Part of the crystal structure of compound (I) showing the formation of a chain of water mol­ecules running parallel to the [100] direction. Hydrogen bonds are drawn as dashed lines, and the H atoms involved in the hydrogen bonds shown have occupancy 0.5. The atoms marked with an asterisk (*), a hash (#), a dollar sign ($) or an ampersand (&) are at the symmetry positions (−*x*, −*y*, −*z*), (*x*, −1 + *y*, −1 + *z*), (1 − *x*, 1 − *y*, 1 − *z*) and (1 + *x*, *y*, *z*), respectively.

**Figure 6:**
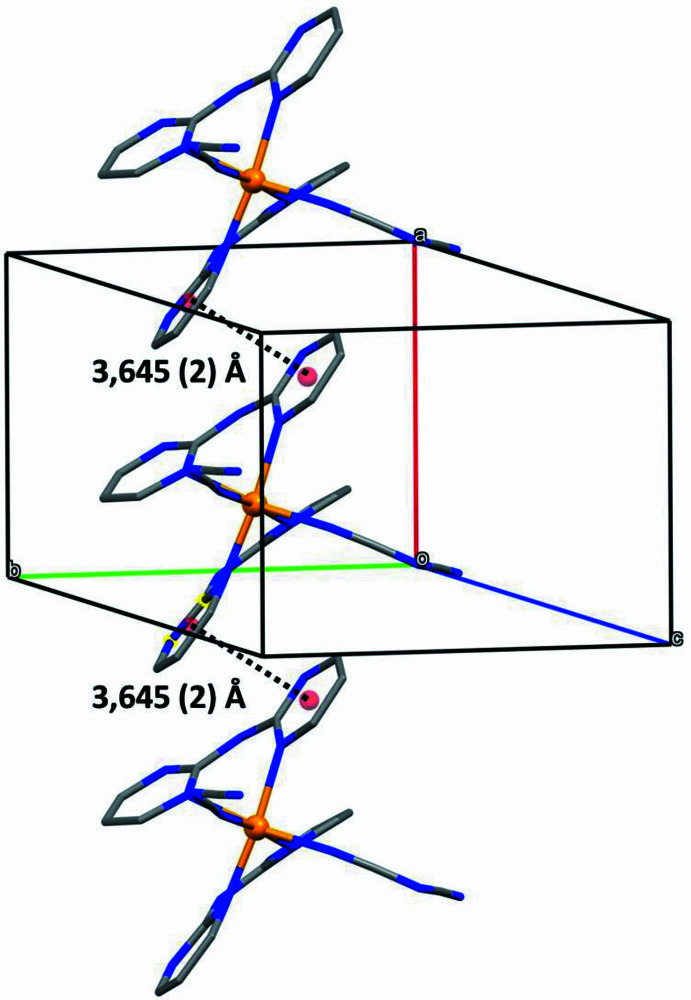
Part of the crystal structure of compound (I) showing the formation of a π-stacked chain running parallel to [100]. For the sake of clarity, the water mol­ecules and the H atoms have all been omitted.

It is possible to identify a number of one-dimensional sub-structures in which the iron complexes are linked into a variety of chains by the water mol­ecules and it is sufficient here to illustrate just two examples, running parallel to the [010] (Fig. 3 ▸) and [001] (Fig. 4 ▸) directions, respectively. It is also possible to identify chains consisting only of water mol­ecules and running along (*x*, 0, 0), (*x*, 0, 1), (*x*, 1, 0) and (*x*, 1, 1) (Fig. 5 ▸). In each of these chains, there are two H-atom sites between successive O atoms (Fig. 5 ▸), with H⋯H distances such that if one of these H sites is occupied, then the other must be vacant, leading to correlation of the H-atom occupancies along the whole chain, consistent with the overall half occupancy of these sites. However, there is no correlation of the H-atom site occupancies between neighbouring chains. The combination of chain motifs along [100], [010] and [001] (Figs. 2 ▸–5 ▸ ▸ ▸) is sufficient to confirm the three-dimensional nature of the hydrogen-bonded assembly, but other chain motifs, in which the iron complexes are linked by water mol­ecules, can be identified running parallel to [110], [011], [ 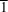 01], [012] and [111]. The two short inter­molecular C—H⋯N contacts both have small *D*—H⋯*A* angles (Table 2 ▸), and so may be of limited structural significance (Wood *et al.*, 2009 ▸). The structure of (I) also contains a single π–π stacking inter­action between iron complexes related by translation along [100]. The rings containing atoms N11 and N31, in the complexes at (*x*, *y*, *z*) and (1 + *x*, *y*, *z*) make a dihedral angle of 11.9 (2)° with a corresponding ring-centroid separation of 3.645 (2) Å, leading to the formation of a weakly π-stacked chain along [100] (Fig. 6 ▸).

We also note the presence of two short inter­molecular anion–π contacts (Table 3 ▸), both involving the terminal N atom of a dicyanamido ligand. Since both these two N atoms also act as acceptors in O—H⋯N hydrogen bonds (Table 2 ▸), it is unclear how significant the anion–π contacts might be.

## 4. Database survey

While there do not appear to be any previous structural reports on iron complexes containing the di(pyrimidin-2-yl)amine ligand, there are a few reports of complexes with other metals, including some coordination polymers involving this ligand bound to copper(II) (Gamez *et al.*, 2005 ▸; van Albada *et al.*, 2007 ▸), and an isostructural pair of mononuclear zinc and cadmium complexes (van Albada *et al.*, 2008 ▸).

## 5. Hirshfeld surface analysis

**Figure 7:**
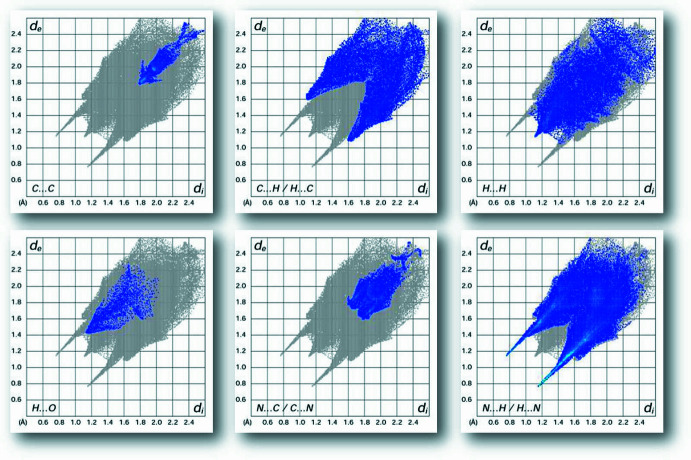
Hirshfeld surface fingerprint plots around the organic molecule.

*MoProViewer* software (Guillot *et al.*, 2014 ▸) was used to investigate the inter­molecular inter­actions and their enrichment on the Hirshfeld surface around the iron complex. The Hirshfeld two-dimensional fingerprint plots of contacts were generated with *Crystal Explorer* (Spackman *et al.*, 2021 ▸). The Hirshfeld contact surface of the iron complex is mainly constituted by C, H-c and N atoms, which represent 95% of the total. The largest contributions for the contacts in the crystal packing are of C—H⋯N and C—H⋯C types, where the C—H⋯N contacts are weak hydrogen bonds. These are followed by the stacking contacts of C⋯C and C⋯N types. In the fingerprint plots (Fig. 7 ▸), there are two short spikes at short distance representing the N⋯H hydrogen bonds. The H⋯H contacts also show a widened spike around the main diagonal at short distances. On the other hand, the iron complex makes C—H⋯O contacts with the water oxygen atoms, at longer distances.

The inter­molecular inter­actions were further evaluated by computing the contact enrichment ratios (supplementary Table 1 ▸) in order to highlight which contacts are favoured. Contacts *X*⋯*Y* that are over-represented with respect to the share of *X* and *Y* chemical species on the Hirshfeld surface have enrichments larger than unity. They are likely to represent attractive inter­actions and thus to be the driving force in the crystal formation (Jelsch *et al.*, 2014 ▸). The enrichment values are obtained as the ratio between the proportions of actual contacts C_*xy*_ and the equiprobable (random) contacts *R_xy_*, the latter being obtained from the probability products (*R_xy_* = *S_x_S_y_*). The strong hydrogen bonds of O—H⋯N and N—H⋯N types represent only 9.2% of the contacts surface but are the most over-represented (*E* = 1.84) among the significant contacts. The abundant C—H⋯N contacts are also quite enriched at *E* = 1.62. Among the major hydro­phobic inter­actions, C⋯C stacking is moderately enriched while the weak C⋯H contacts are marginally under-represented (*E* = 1.27 and 0.94, respectively).

## 6. Synthesis and crystallization

The ligand di(pyrimidin-2-yl)amine (dipm) was prepared according to the published method (Yao *et al.*, 2000 ▸). The title compound was prepared solvothermally under autogenous pressure from a mixture of iron(II) bis­(tetra­fluoro­borate) hexa­hydrate (34 mg, 0.1 mmol), dipm (35 mg, 0.2 mmol) and sodium dicyanamide (18 mg, 0.2 mmol) in a mixture of water and ethanol (4:1 *v*/*v*, 20 ml). This mixture was sealed in a Teflon-lined autoclave and held at 403 K for two days, and then cooled to ambient temperature at a rate of 10 K h^−1^ to give the product (yield 42%). Yellow needle-shaped crystals of the title compound were selected directly from the synthesized product.

## 7. Refinement

Crystal data, data collection and refinement details are summarized in Table 4 ▸. One bad outlier reflection, ( 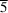 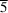 9), was removed from the data set. The refinement was handled as a non-merohedral twin, with twin matrix (1.000, 0.000, 0.000/-0.016, −1.000, 0.000/–0.183, 0.000, −1.000) and with refined twin fractions of 0.178 (3) and 0.822 (3). All the H atoms were located in difference maps. The H atoms bonded to C atoms were then treated as riding atoms in geometrically idealized positions with C—H distances 0.95 Å and with *U*_iso_(H) = 1.2*U*_eq_(C). In each of the water mol­ecules, one of the H atoms was found to be disordered over two atomic sites: when the atomic coordinates of the water H atoms were refined with *U*_iso_(H) = 1.5*U*_eq_(O) but with no geometrical restraints, the resulting O—H distances were closely clustered around 0.86 Å, but the range of the H—O—H angles was too large to be regarded as satisfactory. Hence distance restraints of O—H = 0.86 (2) Å and H⋯H = 1.36 (2) Å were applied to both water mol­ecules. A number of apparently short inter­molecular H⋯H distances indicated strong correlation between the occupancies of the sites H72, H73, H82 and H83, and refinement of these occupancies, subject to such correlation, gave values well within one s.u. of 0.5: consequently these occupancies were all fixed at 0.5. The resulting hydrogen-bond parameters are given in Table 2 ▸. For the H atoms bonded to N atoms or O atoms, the atomic coordinates were refined with *U*_iso_(H) = 1.2*U*_eq_(N) giving N—H distances of 0.84 (4) and 0.89 (5) Å.

## Supplementary Material

Crystal structure: contains datablock(s) global, I. DOI: 10.1107/S2056989023008186/hb8076sup1.cif

Structure factors: contains datablock(s) I. DOI: 10.1107/S2056989023008186/hb8076Isup2.hkl

The proportions of the different contacts and their enrichment in the title compound. DOI: 10.1107/S2056989023008186/hb8076sup3.pdf

CCDC reference: 2295696

Additional supporting information:  crystallographic information; 3D view; checkCIF report

## Figures and Tables

**Table 1:** Selected geometric parameters (Å, °)

Fe1—N61	2.077 (4)	C52—N53	1.301 (6)
Fe1—N51	2.144 (4)	N53—C54	1.321 (6)
Fe1—N11	2.175 (3)	C54—N55	1.156 (6)
Fe1—N31	2.176 (3)	N61—C62	1.151 (5)
Fe1—N41	2.224 (3)	C62—N63	1.297 (6)
Fe1—N21	2.230 (3)	N63—C64	1.310 (6)
N51—C52	1.157 (6)	C64—N65	1.129 (6)
			
N31—Fe1—N41	79.26 (12)	C52—N53—C54	119.7 (4)
N11—Fe1—N21	78.51 (12)	C62—N61—Fe1	170.6 (4)
C52—N51—Fe1	145.4 (3)	C62—N63—C64	121.4 (4)

**Table 2:** Hydrogen-bond geometry (Å, °)

*D*—H⋯*A*	*D*—H	H⋯*A*	*D*⋯*A*	*D*—H⋯*A*
N1—H1⋯N23^i^	0.84 (4)	2.11 (5)	2.942 (4)	170 (5)
N3—H3⋯N43^ii^	0.89 (5)	2.03 (5)	2.918 (4)	175 (5)
O71—H71⋯N55	0.86 (5)	2.11 (5)	2.959 (6)	173 (6)
O71—H72⋯O81^iii^	0.86 (6)	1.84 (5)	2.683 (6)	164 (11)
O71—H73⋯O71^iv^	0.85 (8)	1.99 (7)	2.770 (6)	152 (8)
O81—H81⋯N65	0.85 (4)	2.04 (4)	2.871 (5)	166 (7)
O81—H82⋯O71^iii^	0.86 (7)	1.89 (6)	2.683 (6)	153 (8)
O81—H83⋯O81^v^	0.86 (8)	2.04 (7)	2.751 (5)	140 (8)
C36—H36⋯N53^iii^	0.95	2.49	3.195 (6)	131
C46—H46⋯N65^vi^	0.95	2.53	3.309 (6)	139

Symmetry codes: (i) 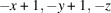 ; (ii) 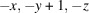 ; (iii) 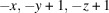 ; (iv) 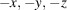 ; (v) 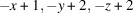 ; (vi) 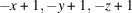 .

**Table 3:** Geometrical parameters (Å, °) for short anion–π contacts *Cg*1 and *Cg*2 represent the centroids of the rings (N31,C32,N33,C34,C35,C36) and (N11,C12,N13,C14,C15,C16), respectively.

C—N⋯*Cg*	C—N	N⋯*Cg*	C⋯*Cg*	C—N⋯*Cg*
C54—N55⋯*Cg*1^i^	1.156 (6)	3.720 (5)	4.469 (6)	123.9 (4)
C64—N65⋯*Cg*2^ii^	1.129 (6)	3.676 (5)	4.049 (5)	101.2 (3)

Symmetry codes: (i) *x*, −1 + *y*, *z*; (ii) 1 − *x*, 1 − *y*, 1 − *z*.

**Table 4:** Experimental details

Crystal data	
Chemical formula	[Fe(C_2_N_3_)_2_(C_8_H_7_N_5_)_2_]·2H_2_O
*M*_r_	570.35
Crystal system, space group	Triclinic, *P* 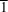
Temperature (K)	170
*a*, *b*, *c* (Å)	8.1960 (7), 10.4671 (11), 14.7926 (14)
α, β, γ (°)	105.254 (4), 92.903 (3), 90.356 (4)
*V* (Å^3^)	1222.5 (2)
*Z*	2
Radiation type	Mo *K*α
μ (mm^−1^)	0.67
Crystal size (mm)	0.25 × 0.20 × 0.15

Data collection	
Diffractometer	Rigaku Oxford Diffraction SuperNova, single source at offset/far, Eos
Absorption correction	Multi-scan (*CrysAlis PRO*; Rigaku OD, 2015 ▸)
*T*_min_, *T*_max_	0.623, 0.906
No. of measured, independent and observed [*I* > 2σ(*I*)] reflections	6056, 6056, 5802
*R*_int_	0.037
(sin θ/λ)_max_ (Å^−1^)	0.668

Refinement	
*R*[*F* ^2^ > 2σ(*F* ^2^)], *wR*(*F* ^2^), *S*	0.067, 0.157, 1.12
No. of reflections	6056
No. of parameters	377
No. of restraints	12
H-atom treatment	H atoms treated by a mixture of independent and constrained refinement
Δρ_max_, Δρ_min_ (e Å^−3^)	1.47, −0.58

Computer programs: *CrysAlis PRO* (Rigaku OD, 2015 ▸), *SHELXS86* (Sheldrick, 2008 ▸), *SHELXL2014* (Sheldrick, 2015 ▸), *PLATON* (Spek, 2020 ▸), *Mercury* (Macrae *et al.*, 2020 ▸) and *publCIF* (Westrip, 2010 ▸).

## supplementary crystallographic information

### Crystal data

**Table:**

[Fe(C_2_N_3_)_2_(C_8_H_7_N_5_)_2_]·2H_2_O	*Z* = 2
*M**_r_* = 570.35	*F*(000) = 584
Triclinic, *P*1	*D*_x_ = 1.549 Mg m^−^^3^
*a* = 8.1960 (7) Å	Mo *K*α radiation, λ = 0.71073 Å
*b* = 10.4671 (11) Å	Cell parameters from 6057 reflections
*c* = 14.7926 (14) Å	θ = 2.5–28.4°
α = 105.254 (4)°	µ = 0.67 mm^−^^1^
β = 92.903 (3)°	*T* = 170 K
γ = 90.356 (4)°	Needle, yellow
*V* = 1222.5 (2) Å^3^	0.25 × 0.20 × 0.15 mm

### Data collection

**Table:**

Rigaku Oxford Diffraction SuperNova, single source at offset/far, Eos diffractometer	6056 independent reflections
Radiation source: SuperNova (Mo) X-ray Source	5802 reflections with *I* > 2σ(*I*)
Mirror monochromator	*R*_int_ = 0.037
ω scans	θ_max_ = 28.4°, θ_min_ = 2.5°
Absorption correction: multi-scan (CrysAlis PRO; Rigaku OD, 2015)	*h* = −10→10
*T*_min_ = 0.623, *T*_max_ = 0.906	*k* = −13→13
6056 measured reflections	*l* = −19→19

### Refinement

**Table:**

Refinement on *F*^2^	Primary atom site location: difference Fourier map
Least-squares matrix: full	Hydrogen site location: mixed
*R*[*F*^2^ > 2σ(*F*^2^)] = 0.067	H atoms treated by a mixture of independent and constrained refinement
*wR*(*F*^2^) = 0.157	*w* = 1/[σ^2^(*F*_o_^2^) + 5.9338*P*] where *P* = (*F*_o_^2^ + 2*F*_c_^2^)/3
*S* = 1.12	(Δ/σ)_max_ < 0.001
6056 reflections	Δρ_max_ = 1.47 e Å^−^^3^
377 parameters	Δρ_min_ = −0.58 e Å^−^^3^
12 restraints	

### Special details

**Table:**

Geometry. All esds (except the esd in the dihedral angle between two l.s. planes) are estimated using the full covariance matrix. The cell esds are taken into account individually in the estimation of esds in distances, angles and torsion angles; correlations between esds in cell parameters are only used when they are defined by crystal symmetry. An approximate (isotropic) treatment of cell esds is used for estimating esds involving l.s. planes.
Refinement. Refined as a 2-component twin.

### Fractional atomic coordinates and isotropic or equivalent isotropic displacement parameters (Å^2^)

**Table:**

	*x*	*y*	*z*	*U*_iso_*/*U*_eq_	Occ. (<1)
Fe1	0.28878 (7)	0.59497 (5)	0.32111 (3)	0.01841 (13)	
N1	0.5194 (4)	0.5167 (3)	0.1355 (2)	0.0228 (7)	
H1	0.547 (6)	0.478 (5)	0.081 (3)	0.027*	
N11	0.5331 (4)	0.5184 (3)	0.2957 (2)	0.0219 (6)	
C12	0.5903 (5)	0.4757 (4)	0.2095 (3)	0.0215 (7)	
N13	0.7127 (4)	0.3911 (3)	0.1857 (2)	0.0278 (7)	
C14	0.7890 (5)	0.3528 (4)	0.2551 (3)	0.0306 (9)	
H14	0.8742	0.2908	0.2405	0.037*	
C15	0.7495 (5)	0.3994 (4)	0.3485 (3)	0.0306 (9)	
H15	0.8100	0.3757	0.3979	0.037*	
C16	0.6190 (5)	0.4812 (4)	0.3648 (3)	0.0286 (8)	
H16	0.5870	0.5134	0.4275	0.034*	
N21	0.3681 (4)	0.6974 (3)	0.2158 (2)	0.0202 (6)	
C22	0.4278 (5)	0.6255 (3)	0.1357 (3)	0.0196 (7)	
N23	0.4029 (4)	0.6478 (3)	0.0507 (2)	0.0262 (7)	
C24	0.3213 (6)	0.7560 (4)	0.0474 (3)	0.0312 (9)	
H24	0.2981	0.7733	−0.0118	0.037*	
C25	0.2687 (6)	0.8452 (4)	0.1280 (3)	0.0290 (8)	
H25	0.2186	0.9259	0.1257	0.035*	
C26	0.2932 (5)	0.8103 (4)	0.2110 (3)	0.0241 (8)	
H26	0.2561	0.8676	0.2669	0.029*	
N3	0.0205 (4)	0.5704 (3)	0.1378 (2)	0.0221 (6)	
H3	−0.026 (6)	0.586 (5)	0.086 (3)	0.027*	
N31	0.0422 (4)	0.6630 (3)	0.3022 (2)	0.0233 (7)	
C32	−0.0328 (5)	0.6564 (4)	0.2185 (3)	0.0208 (7)	
N33	−0.1591 (4)	0.7287 (3)	0.2022 (2)	0.0278 (7)	
C34	−0.2224 (5)	0.8094 (4)	0.2778 (3)	0.0309 (9)	
H34	−0.3109	0.8639	0.2692	0.037*	
C35	−0.1622 (6)	0.8155 (5)	0.3684 (3)	0.0344 (10)	
H35	−0.2121	0.8683	0.4218	0.041*	
C36	−0.0275 (5)	0.7417 (4)	0.3772 (3)	0.0294 (9)	
H36	0.0184	0.7461	0.4382	0.035*	
N41	0.1913 (4)	0.4328 (3)	0.2006 (2)	0.0206 (6)	
C42	0.1115 (5)	0.4589 (4)	0.1268 (2)	0.0198 (7)	
N43	0.1116 (4)	0.3831 (3)	0.0374 (2)	0.0253 (7)	
C44	0.1914 (6)	0.2702 (4)	0.0234 (3)	0.0289 (9)	
H44	0.1978	0.2163	−0.0389	0.035*	
C45	0.2662 (5)	0.2281 (4)	0.0969 (3)	0.0267 (8)	
H45	0.3150	0.1437	0.0868	0.032*	
C46	0.2661 (5)	0.3141 (4)	0.1844 (3)	0.0211 (7)	
H46	0.3202	0.2900	0.2356	0.025*	
N51	0.2329 (5)	0.4644 (4)	0.4057 (2)	0.0326 (8)	
C52	0.1779 (5)	0.3624 (4)	0.4059 (3)	0.0276 (8)	
N53	0.1117 (7)	0.2525 (4)	0.4132 (3)	0.0489 (12)	
C54	0.0792 (6)	0.1549 (5)	0.3370 (3)	0.0356 (10)	
N55	0.0436 (6)	0.0672 (4)	0.2730 (3)	0.0485 (11)	
N61	0.3620 (5)	0.7507 (4)	0.4355 (2)	0.0338 (8)	
C62	0.4141 (6)	0.8241 (4)	0.5033 (3)	0.0326 (9)	
N63	0.4727 (9)	0.9156 (5)	0.5750 (3)	0.0694 (19)	
C64	0.4855 (6)	0.8965 (4)	0.6591 (3)	0.0325 (9)	
N65	0.5029 (6)	0.8940 (4)	0.7348 (3)	0.0404 (9)	
O71	−0.0803 (5)	0.0590 (4)	0.0801 (3)	0.0548 (10)	
H71	−0.037 (8)	0.059 (7)	0.134 (3)	0.082*	
H72	−0.177 (6)	0.025 (11)	0.079 (6)	0.082*	0.5
H73	−0.028 (11)	0.002 (9)	0.041 (4)	0.082*	0.5
O81	0.4001 (5)	0.9987 (4)	0.9222 (2)	0.0492 (10)	
H81	0.439 (8)	0.981 (7)	0.868 (2)	0.074*	
H82	0.309 (7)	0.955 (9)	0.915 (6)	0.074*	0.5
H83	0.464 (9)	0.960 (9)	0.954 (5)	0.074*	0.5

### Atomic displacement parameters (Å^2^)

**Table:**

	*U*^11^	*U*^22^	*U*^33^	*U*^12^	*U*^13^	*U*^23^
Fe1	0.0221 (3)	0.0223 (3)	0.0094 (2)	0.0008 (2)	−0.00059 (18)	0.00175 (18)
N1	0.0282 (17)	0.0237 (16)	0.0160 (14)	0.0051 (13)	0.0043 (12)	0.0041 (12)
N11	0.0203 (15)	0.0290 (16)	0.0161 (14)	−0.0020 (12)	−0.0004 (11)	0.0055 (12)
C12	0.0218 (17)	0.0214 (17)	0.0211 (17)	0.0000 (14)	0.0009 (14)	0.0051 (14)
N13	0.0311 (18)	0.0271 (17)	0.0277 (17)	0.0068 (14)	0.0048 (14)	0.0108 (14)
C14	0.027 (2)	0.032 (2)	0.036 (2)	0.0074 (17)	0.0041 (17)	0.0158 (18)
C15	0.027 (2)	0.037 (2)	0.031 (2)	0.0004 (17)	−0.0056 (16)	0.0145 (18)
C16	0.030 (2)	0.038 (2)	0.0184 (17)	−0.0032 (17)	−0.0054 (15)	0.0086 (16)
N21	0.0252 (16)	0.0181 (14)	0.0165 (14)	−0.0010 (12)	0.0024 (12)	0.0027 (11)
C22	0.0225 (17)	0.0163 (16)	0.0199 (16)	−0.0033 (13)	0.0034 (13)	0.0044 (13)
N23	0.0364 (19)	0.0249 (16)	0.0193 (15)	0.0050 (14)	0.0054 (13)	0.0088 (13)
C24	0.046 (3)	0.026 (2)	0.026 (2)	0.0066 (18)	0.0070 (18)	0.0135 (16)
C25	0.040 (2)	0.0185 (17)	0.032 (2)	0.0050 (16)	0.0084 (18)	0.0099 (15)
C26	0.030 (2)	0.0152 (16)	0.0243 (18)	−0.0006 (14)	0.0052 (15)	0.0005 (14)
N3	0.0288 (17)	0.0225 (15)	0.0128 (13)	0.0029 (13)	−0.0043 (12)	0.0020 (12)
N31	0.0244 (16)	0.0262 (16)	0.0161 (14)	0.0041 (13)	0.0018 (12)	−0.0004 (12)
C32	0.0222 (17)	0.0200 (17)	0.0200 (16)	0.0016 (14)	0.0008 (14)	0.0051 (13)
N33	0.0292 (18)	0.0227 (16)	0.0276 (17)	0.0056 (14)	−0.0048 (14)	0.0005 (13)
C34	0.025 (2)	0.027 (2)	0.037 (2)	0.0053 (16)	−0.0007 (17)	0.0029 (17)
C35	0.033 (2)	0.037 (2)	0.029 (2)	0.0061 (18)	0.0088 (17)	0.0004 (18)
C36	0.034 (2)	0.033 (2)	0.0193 (18)	0.0025 (17)	0.0056 (16)	0.0026 (16)
N41	0.0249 (16)	0.0199 (15)	0.0167 (14)	−0.0003 (12)	0.0005 (12)	0.0044 (11)
C42	0.0245 (18)	0.0181 (16)	0.0154 (15)	−0.0042 (13)	−0.0021 (13)	0.0029 (13)
N43	0.0369 (19)	0.0203 (15)	0.0157 (14)	0.0020 (13)	−0.0047 (13)	0.0007 (12)
C44	0.045 (2)	0.0206 (18)	0.0182 (17)	0.0039 (17)	−0.0035 (16)	0.0005 (14)
C45	0.037 (2)	0.0200 (17)	0.0225 (18)	0.0045 (16)	−0.0027 (16)	0.0061 (14)
C46	0.0248 (18)	0.0201 (17)	0.0200 (16)	−0.0005 (14)	−0.0015 (14)	0.0087 (14)
N51	0.044 (2)	0.038 (2)	0.0158 (15)	−0.0006 (17)	0.0008 (14)	0.0076 (14)
C52	0.035 (2)	0.034 (2)	0.0154 (16)	0.0064 (17)	0.0033 (15)	0.0094 (15)
N53	0.083 (4)	0.041 (2)	0.0258 (19)	−0.007 (2)	0.013 (2)	0.0110 (17)
C54	0.035 (2)	0.035 (2)	0.040 (2)	0.0089 (19)	0.0094 (19)	0.014 (2)
N55	0.052 (3)	0.039 (2)	0.049 (3)	0.002 (2)	0.006 (2)	0.002 (2)
N61	0.043 (2)	0.0331 (19)	0.0202 (16)	−0.0012 (16)	−0.0024 (15)	−0.0008 (14)
C62	0.048 (3)	0.027 (2)	0.0219 (19)	−0.0028 (19)	−0.0040 (18)	0.0072 (16)
N63	0.142 (6)	0.037 (2)	0.025 (2)	−0.033 (3)	−0.023 (3)	0.0074 (18)
C64	0.044 (3)	0.0227 (19)	0.025 (2)	−0.0026 (18)	−0.0047 (18)	−0.0018 (16)
N65	0.050 (3)	0.040 (2)	0.0263 (19)	−0.0017 (19)	−0.0038 (17)	0.0014 (16)
O71	0.046 (2)	0.066 (3)	0.048 (2)	−0.002 (2)	0.0036 (18)	0.005 (2)
O81	0.042 (2)	0.070 (3)	0.0287 (17)	0.0037 (19)	0.0054 (15)	−0.0011 (17)

### Geometric parameters (Å, º)

**Table:**

Fe1—N61	2.077 (4)	N31—C36	1.350 (5)
Fe1—N51	2.144 (4)	C32—N33	1.335 (5)
Fe1—N11	2.175 (3)	N33—C34	1.342 (5)
Fe1—N31	2.176 (3)	C34—C35	1.388 (6)
Fe1—N41	2.224 (3)	C34—H34	0.9500
Fe1—N21	2.230 (3)	C35—C36	1.372 (6)
N1—C22	1.367 (5)	C35—H35	0.9500
N1—C12	1.381 (5)	C36—H36	0.9500
N1—H1	0.84 (5)	N41—C42	1.335 (5)
N11—C12	1.344 (5)	N41—C46	1.357 (5)
N11—C16	1.354 (5)	C42—N43	1.351 (4)
C12—N13	1.341 (5)	N43—C44	1.327 (5)
N13—C14	1.327 (5)	C44—C45	1.394 (5)
C14—C15	1.394 (6)	C44—H44	0.9500
C14—H14	0.9500	C45—C46	1.368 (5)
C15—C16	1.365 (6)	C45—H45	0.9500
C15—H15	0.9500	C46—H46	0.9500
C16—H16	0.9500	N51—C52	1.157 (6)
N21—C22	1.343 (5)	C52—N53	1.301 (6)
N21—C26	1.352 (5)	N53—C54	1.321 (6)
C22—N23	1.343 (5)	C54—N55	1.156 (6)
N23—C24	1.330 (5)	N61—C62	1.151 (5)
C24—C25	1.396 (6)	C62—N63	1.297 (6)
C24—H24	0.9500	N63—C64	1.310 (6)
C25—C26	1.375 (6)	C64—N65	1.129 (6)
C25—H25	0.9500	O71—H71	0.860 (19)
C26—H26	0.9500	O71—H72	0.86 (2)
N3—C42	1.367 (5)	O71—H73	0.85 (2)
N3—C32	1.385 (5)	O81—H81	0.858 (19)
N3—H3	0.89 (5)	O81—H82	0.86 (2)
N31—C32	1.340 (5)	O81—H83	0.86 (2)
			
N61—Fe1—N51	93.91 (15)	C42—N3—C32	130.2 (3)
N61—Fe1—N11	95.09 (14)	C42—N3—H3	117 (3)
N51—Fe1—N11	93.49 (14)	C32—N3—H3	112 (3)
N61—Fe1—N31	96.63 (14)	C32—N31—C36	115.9 (3)
N51—Fe1—N31	97.71 (14)	C32—N31—Fe1	124.0 (3)
N11—Fe1—N31	163.16 (11)	C36—N31—Fe1	118.5 (3)
N61—Fe1—N41	175.73 (15)	N33—C32—N31	126.1 (3)
N51—Fe1—N41	85.54 (13)	N33—C32—N3	113.3 (3)
N11—Fe1—N41	89.17 (12)	N31—C32—N3	120.6 (3)
N31—Fe1—N41	79.26 (12)	C32—N33—C34	116.4 (4)
N61—Fe1—N21	94.04 (13)	N33—C34—C35	122.0 (4)
N51—Fe1—N21	169.19 (13)	N33—C34—H34	119.0
N11—Fe1—N21	78.51 (12)	C35—C34—H34	119.0
N31—Fe1—N21	88.64 (12)	C36—C35—C34	116.9 (4)
N41—Fe1—N21	87.07 (11)	C36—C35—H35	121.5
C22—N1—C12	130.0 (3)	C34—C35—H35	121.5
C22—N1—H1	112 (3)	N31—C36—C35	122.4 (4)
C12—N1—H1	117 (3)	N31—C36—H36	118.8
C12—N11—C16	115.6 (3)	C35—C36—H36	118.8
C12—N11—Fe1	123.4 (3)	C42—N41—C46	116.1 (3)
C16—N11—Fe1	119.1 (3)	C42—N41—Fe1	121.2 (2)
N13—C12—N11	125.8 (4)	C46—N41—Fe1	117.7 (2)
N13—C12—N1	114.0 (3)	N41—C42—N43	125.5 (3)
N11—C12—N1	120.2 (3)	N41—C42—N3	120.4 (3)
C14—N13—C12	116.3 (4)	N43—C42—N3	114.2 (3)
N13—C14—C15	122.9 (4)	C44—N43—C42	116.7 (3)
N13—C14—H14	118.5	N43—C44—C45	122.3 (4)
C15—C14—H14	118.5	N43—C44—H44	118.9
C16—C15—C14	116.1 (4)	C45—C44—H44	118.9
C16—C15—H15	121.9	C46—C45—C44	116.7 (4)
C14—C15—H15	121.9	C46—C45—H45	121.6
N11—C16—C15	122.9 (4)	C44—C45—H45	121.6
N11—C16—H16	118.5	N41—C46—C45	122.3 (3)
C15—C16—H16	118.5	N41—C46—H46	118.8
C22—N21—C26	115.9 (3)	C45—C46—H46	118.8
C22—N21—Fe1	119.2 (2)	C52—N51—Fe1	145.4 (3)
C26—N21—Fe1	118.4 (2)	N51—C52—N53	175.0 (4)
N23—C22—N21	125.8 (3)	C52—N53—C54	119.7 (4)
N23—C22—N1	114.0 (3)	N55—C54—N53	176.1 (6)
N21—C22—N1	120.2 (3)	C62—N61—Fe1	170.6 (4)
C24—N23—C22	116.6 (3)	N61—C62—N63	174.3 (5)
N23—C24—C25	122.2 (4)	C62—N63—C64	121.4 (4)
N23—C24—H24	118.9	N65—C64—N63	172.4 (5)
C25—C24—H24	118.9	H71—O71—H72	105 (3)
C26—C25—C24	116.7 (4)	H71—O71—H73	106 (3)
C26—C25—H25	121.7	H72—O71—H73	106 (3)
C24—C25—H25	121.7	H81—O81—H82	105 (3)
N21—C26—C25	122.3 (3)	H81—O81—H83	104 (3)
N21—C26—H26	118.8	H82—O81—H83	105 (3)
C25—C26—H26	118.8		
			
C16—N11—C12—N13	6.7 (6)	C36—N31—C32—N33	6.5 (6)
Fe1—N11—C12—N13	−157.3 (3)	Fe1—N31—C32—N33	−158.9 (3)
C16—N11—C12—N1	−174.9 (4)	C36—N31—C32—N3	−174.7 (4)
Fe1—N11—C12—N1	21.1 (5)	Fe1—N31—C32—N3	19.8 (5)
C22—N1—C12—N13	−159.5 (4)	C42—N3—C32—N33	−159.9 (4)
C22—N1—C12—N11	21.9 (6)	C42—N3—C32—N31	21.2 (6)
N11—C12—N13—C14	−4.0 (6)	N31—C32—N33—C34	−4.5 (6)
N1—C12—N13—C14	177.5 (4)	N3—C32—N33—C34	176.7 (4)
C12—N13—C14—C15	−1.8 (6)	C32—N33—C34—C35	−1.3 (6)
N13—C14—C15—C16	4.3 (7)	N33—C34—C35—C36	4.2 (7)
C12—N11—C16—C15	−3.7 (6)	C32—N31—C36—C35	−3.0 (6)
Fe1—N11—C16—C15	161.0 (3)	Fe1—N31—C36—C35	163.3 (4)
C14—C15—C16—N11	−1.3 (6)	C34—C35—C36—N31	−2.0 (7)
C26—N21—C22—N23	−8.3 (6)	C46—N41—C42—N43	−6.1 (6)
Fe1—N21—C22—N23	143.1 (3)	Fe1—N41—C42—N43	148.2 (3)
C26—N21—C22—N1	173.6 (3)	C46—N41—C42—N3	174.3 (3)
Fe1—N21—C22—N1	−35.0 (5)	Fe1—N41—C42—N3	−31.4 (5)
C12—N1—C22—N23	168.6 (4)	C32—N3—C42—N41	−14.0 (6)
C12—N1—C22—N21	−13.1 (6)	C32—N3—C42—N43	166.4 (4)
N21—C22—N23—C24	5.0 (6)	N41—C42—N43—C44	3.6 (6)
N1—C22—N23—C24	−176.8 (4)	N3—C42—N43—C44	−176.8 (4)
C22—N23—C24—C25	2.2 (7)	C42—N43—C44—C45	2.6 (6)
N23—C24—C25—C26	−5.3 (7)	N43—C44—C45—C46	−5.5 (7)
C22—N21—C26—C25	4.6 (6)	C42—N41—C46—C45	2.7 (6)
Fe1—N21—C26—C25	−147.1 (3)	Fe1—N41—C46—C45	−152.6 (3)
C24—C25—C26—N21	1.7 (6)	C44—C45—C46—N41	2.7 (6)

### Hydrogen-bond geometry (Å, º)

**Table:**

*D*—H···*A*	*D*—H	H···*A*	*D*···*A*	*D*—H···*A*
N1—H1···N23^i^	0.84 (4)	2.11 (5)	2.942 (4)	170 (5)
N3—H3···N43^ii^	0.89 (5)	2.03 (5)	2.918 (4)	175 (5)
O71—H71···N55	0.86 (5)	2.11 (5)	2.959 (6)	173 (6)
O71—H72···O81^iii^	0.86 (6)	1.84 (5)	2.683 (6)	164 (11)
O71—H73···O71^iv^	0.85 (8)	1.99 (7)	2.770 (6)	152 (8)
O81—H81···N65	0.85 (4)	2.04 (4)	2.871 (5)	166 (7)
O81—H82···O71^iii^	0.86 (7)	1.89 (6)	2.683 (6)	153 (8)
O81—H83···O81^v^	0.86 (8)	2.04 (7)	2.751 (5)	140 (8)
C36—H36···N53^iii^	0.95	2.49	3.195 (6)	131
C46—H46···N65^vi^	0.95	2.53	3.309 (6)	139

Symmetry codes: (i) −*x*+1, −*y*+1, −*z*; (ii) −*x*, −*y*+1, −*z*; (iii) −*x*, −*y*+1, −*z*+1; (iv) −*x*, −*y*, −*z*; (v) −*x*+1, −*y*+2, −*z*+2; (vi) −*x*+1, −*y*+1, −*z*+1.
