# Supplementary material for: Racemic cis-bis­[bis­(pyrimidin-2-yl)amine-κN]bis(dicyanamido-κN 1)iron(II) dihydrate: synthesis, crystal structure and Hirshfeld surface analysis
Source: Acta Crystallogr E Crystallogr Commun. 2023 Sep 26;79(Pt 10):936–41. doi: 10.1107/S2056989023008186 (PMC10561211; doi:10.1107/S2056989023008186)
Supplement: Supplementary file 3 [file e-79-00936-sup3.pdf]

**Atom type**	Ho/n	C	N	O	Fe	H-c
Surface_int (%)	4.18	37.68	26.48	0.00	0.78	30.89
Surface_ext (%)	15.17	33.98	23.84	3.26	0.80	22.95
Contacts (%)						
Ho/n	0.6					
C	3.6	**16.3**				
N	**9.2**	**15.2**	2.0			
O	0.0	1.2	0.0	0.0		
Fe	0.0	1.0	0.0	0.0	0.0	
H-c	5.3	**18.0**	**21.8**	2.0	0.5	3.1
Enrichment						
Ho/n	0.95					
C	0.50	**1.27**				
N	**1.84**	0.85	0.32			
O	0.00	1.00	0.00	/		
H-c	0.95	0.94	**1.62**	2.02	1.22	0.43
